# Plant-Based Functional Foods from Borneo

**DOI:** 10.3390/nu17020200

**Published:** 2025-01-07

**Authors:** Oliver Dean John, Noumie Surugau, Jibrail Kansedo, Sunil K. Panchal, Lindsay Brown

**Affiliations:** 1 Nutritional Biochemistry Research Group, Faculty of Food Science and Nutrition, Universiti Malaysia Sabah, Kota Kinabalu 88400, Sabah, Malaysia; odjohn@ums.edu.my; 2 Seaweed Research Unit, Industrial Chemistry Program, Faculty of Science and Natural Resources, Universiti Malaysia Sabah, Kota Kinabalu 88400, Sabah, Malaysia; lnoumie@ums.edu.my; 3 Department of Chemical and Energy Engineering, Faculty of Engineering and Science, Curtin University Malaysia, CDT 250, Miri 98009, Sarawak, Malaysia; jibrail.k@curtin.edu.my; 4 School of Science, Western Sydney University, Hawkesbury Campus, Richmond, NSW 2753, Australia; s.panchal@westernsydney.edu.au; 5 School of Pharmacy and Medical Sciences, Griffith University, Gold Coast, QLD 4222, Australia

**Keywords:** Borneo, functional foods, plant-based foods, Sabah, Sarawak, Brunei, Kalimantan, Indonesia, Malaysia, healthy diet

## Abstract

Borneo, the third-largest island in the world, is shared between Malaysia (Sabah and Sarawak), Indonesia (Kalimantan) and Brunei. As a biodiversity hotspot, it is home to about 15,000 flowering plants and 3000 tree species, of which many are endemic to the region. Locally derived plant-based foods are gaining popularity due to their lower environmental impact, contribution to food sustainability and health benefits. The local fruits and vegetables of Borneo have been used traditionally by the indigenous community for medicinal purposes. This community knowledge can provide a valuable guide to their potential for use as functional foods. This review explores the contemporary foods from Borneo, including fruit, vegetables, seaweeds and plant-derived food products that are locally consumed. The findings show that the unique tropical food groups have a wide diversity of phytochemical compositions that possess a wide array of biological activities including anti-inflammatory, antioxidant, anti-microbial, anti-proliferative, anti-fungal, wound healing and expectorant properties. The wide range of plant-based foods in Borneo deserves further development for wider applications as functional foods.

## 1. Introduction

Borneo is the third-largest island in the world after Greenland and New Guinea [1,2] and is shared by three countries, Indonesia (Kalimantan), Malaysia (Borneo states of Sarawak and Sabah) and Brunei Darussalam [3]. Although the rainforests in Borneo cover only about 1% of the earth’s terrestrial surface, they hold approximately 6% of the world’s animal and plant species [4]. As home to one of the planet’s oldest rainforests [5], Borneo’s forests harbour approximately 15,000 flowering plants and 3000 species of trees, many of which are unique to Borneo, thus making this island a globally important biodiversity hotspot [4]. The Kalimantan region contributes the largest forest area to the Borneo region, having the most land mass, and it is rich in animal and plant biodiversity [4]. Sarawak is the largest Malaysian state, located on the northwest of Borneo [6], with tropical rainforest covering about 8 million hectares. Sabah’s diverse habitats include mountain forests, rivers, flood plains and the surrounding seas, which provide dynamic interactions between flora and fauna. Despite having the smallest land area, Brunei is known for its extensive forest preservation, avoiding large-scale logging and oil palm plantation. About 54% of its land area remains covered by an intact forest ecosystem, significantly conserving the diverse flora and fauna in this region [7]. These ecosystems, alongside the indigenous communities’ deep knowledge of animal and plant diversity, ecological relationships and seasonal rhythms, enable the sustainable extraction of food, medicine and raw materials [8,9].

The term “plant-based diet” was first introduced by Dr. Thomas Collin Campbell in 1980 [10]. While the whole definition of plant-based diet remains to be properly defined [11], a plant-based diet consists of higher intakes of plant foods such as vegetables, fruits, whole grains, legumes, seeds and nuts while excluding animal products [12]. Further, macroalgae such as seaweeds provide important nutrients such as vitamins, minerals and phytochemicals including phenolic acids and flavonoids [13], while microalgae are increasingly used for providing protein and other nutrients [14] that could be incorporated into the plant-based food category [15].

Plant-based foods are increasingly gaining more exposure due to their valuable impacts towards human health, environmental sustainability and global food security [16]. In the UK, for example, the plant-based alternative foods consumption rose by 115% in 2017–2019 compared with 2008–2011 [17]. The production of plant-based foods requires less energy and fewer natural resources compared with animal-based foods. On average, about 11 times more fossil energy is needed to produce animal protein than plant protein used for human consumption [18]. For instance, when considering water consumption, producing 1 kg of beef requires about 13,000 litres of water, but only 1250 litres of water are required to produce 1 kg of lentils [19]. The practice of lacto-ovo-vegetarian diet can reduce freshwater use by 28%, greenhouse gas emissions by 35% and land use by 42% [20]. The increased adoption of plant-based diets is also due to their perceived health benefits. The consumption of plant-based diets has been correlated with reduced risk of obesity, type 2 diabetes, cardiovascular diseases, hypertension, hypercholesterolaemia and cancer [21,22]. A plant-based diet also supports the maintenance of a healthy gut microbiome [23].

The diets of Borneo’s native people are distinguished by a wide array of plant-based foods that not only provide energy but are also shaped by the people’s cultural identity and ecological knowledge. These foods include a variety of leafy greens, roots, tubers and fruits that are rich in vitamins, minerals and phytonutrients. The use of plant-based diets as functional foods has been widely discussed in the literature [24,25]. Recently, we have discussed the potential of tropical foods in modulating the symptoms of metabolic syndrome [26] and the potential of anthocyanin-containing foods and their interactions with several disorders [27]. This review aims to explore the plant-based foods from Borneo and discuss their health benefits towards the application in wider populations. The wild fruits of Borneo have been documented [28]. However, this review aims to emphasise the local fruits and vegetables that are currently cultivated and mostly native to Borneo and foods that are under-reported in the literature.

## 2. Tropical Fruits

Fruits are an essential part of a healthy diet and provide important sources of macronutrients and micronutrients such as fibre, vitamins and minerals together with other bioactive phytochemicals, especially antioxidants [29]. Recent reviews have shown that tropical fruits have beneficial effects against chronic and metabolic diseases [26,30,31]. Some wild plants and fruits serve as a source of food in the diet of local communities [32]. Indigenous tropical fruits have long been cultivated for solely personal or community consumption. However, due to increased demand, large-scale farming has been introduced lately for commercialisation [33]. For instance, the Dayak community in Kalimantan has practiced traditional medicine using medicinal plants, which have been passed down for generations through oral methods [34]. Among the reported traditional knowledge of fruits are the use of soursop (*Annona muricata*) fruits to treat high blood pressure, gout, back pain and rheumatism; papaya (*Carica papaya*) for fever, intestinal worms and headache; and mangosteen (*Garcinia mangostana*) for treating headaches and high blood cholesterol [34].

### 2.1. Durians

**Figure 1:**
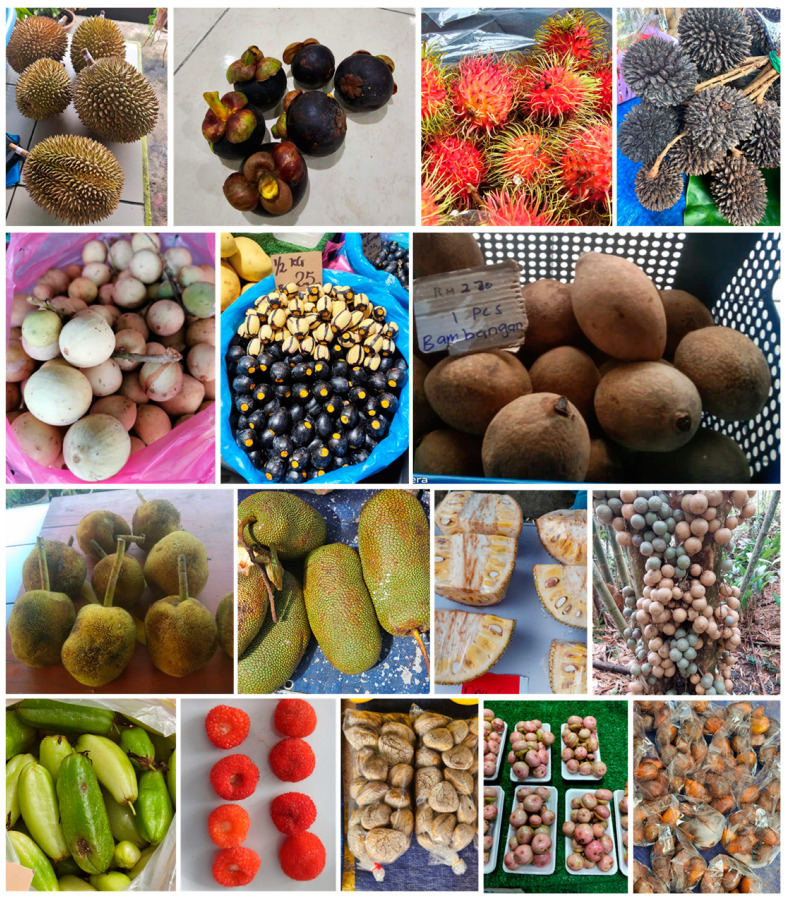
Tropical fruits from Borneo. First row—(left to right) durian (*Durio* spp.), purple mangosteen (*Garcinia mangostana*), rambutan (*Nephelium lappaceum*), pulasan (*Nephelium ramboutan-ake*); second row—(left to right) langsat (*Lansium domesticum*), dabai (*Canarium odontophyllum*), bambangan (*Mangifera pajang*); third row—(left to right) tarap (*Artocarpus odoratissimus*), cempedak (*Artocarpus integer*), jackfruit (*Artocarpus heterophyllus*), liposu (*Baccaurea lanceolata*); fourth row—belimbing buluh (*Averrhoa bilimbi*), wild berries (*Rubus rosifolius*), pangi (*Pangium edule*), pengolaban (*Litsea garciae*), salak (*Salacca* sp.).

Durians (*Durio* spp.) (Figure 1), dubbed as the “king of fruits”, are popular for their distinctive smell and taste [35] and their thorny appearance [36]. There are 30 species described in the literature, but only about a third produce edible fruits [35]. The pulp is eaten fresh, while the seeds may be roasted and boiled; the flowers and unripe flesh are also eaten in curries [37,38]. The fresh fruits are fermented to make a traditional delicacy named “tempoyak” [35]. Borneo is regarded as the centre of diversity of durians with 15 endemic species [35]. In Sarawak alone, there are 16 unique species of durians, but only half are edible, including *D. dulcis*, *D. graveolens*, *D. gradiflorus*, *D. kutejensis*, *D. lowianus*, *D. testudinarum*, *D. oxleyanus* and *D. zibethinus*, with *D. zibethinus* being widely cultivated [36]. A genetic analysis of the wild durians from Sarawak showed that they are genetically varied even within identical species. The wild durians are popular among locals and are unique compared with the commercially available *D. zibethinus* variety, with different appearances (long, sharp thorns, distinct flesh colours of yellow and orange, and a red pericarp colour) and unique tastes. These wild edible durians have important bioactive compounds and exotic tastes along with valuable nutritional compositions. The total sugar and sucrose contents of *D. graveolens* fruits are lower compared with the other species, thus contributing to their bland taste; they are usually made into “tempoyak”. However, *D. graveolens* fruits have almost twice the amount of fat content compared with the rest of the fruits and greater fibre content. The indigenous durians are also an important source of minerals such as K, P, Mg and trace minerals. *D. graveolens* and *D. kutejensis* are important antioxidants due to their high ascorbic acid and carotenoid content, while *D. dulcis* and *D. oxleyanus* contain plentiful flavonoids and phenolic compounds, which are reported to be essential to maintaining health and alleviating chronic disease [36].

Sabah is also home to 14 of these species. *D. kinabaluensis*, commonly known as “durian tupoloh”, is adapted to high altitudes and typically grows from the foothills of the Crocker Range to the slopes of Mount Kinabalu, primarily outside the park boundaries. This species, also referred to as “durian kinabalu,” is endemic to Sabah, and its nutritional and health benefits have yet to be documented [39]. Meanwhile, *D. oxleyanus*, called “durian sukang” in Sabah and “kerantongan” in Indonesia, is found in Borneo, Peninsular Malaysia and Sumatra. This species thrives in lowland mixed dipterocarp forests [40]. The phytochemical compositions and antioxidant properties of different durian parts have been investigated for both *D. kinabaluensis* and *D. oxleyanus*, showing high flavonoid content in the exocarp and seed of *D. oxleyanus* while reporting high total phenolic and total flavonoid contents in the mesocarp of *D. kinabaluensis* [39]. In general, the inedible parts of both durians showed higher phytochemical and antioxidant activities, showing the potential of these parts for generating nutraceuticals [39].

### 2.2. Purple Mangosteen and Other Garcinia Fruits

*Garcinia* fruits are cultivated predominantly in tropical regions, and the genus comprises around 250 species throughout the world. In Borneo, the seasonal purple mangosteen, *G. mangostana* (Figure 1), is among the most popular species. The fruit is characterised by its purple rind and white flesh that is sweet and slightly acidic. The flesh is usually eaten as dessert on its own or with other local fruits, with the seed and peel usually discarded. Traditionally, the rind has been used for skin diseases, wounds, to relieve diarrhoea and to aid digestion [41]. The rind contains α-mangostin, which is a xanthone compound reported to have many positive biological effects including anti-inflammatory, anti-diabetic and anti-obesity [41]. The supplementation of purple mangosteen rind powder in the obese rat model of human metabolic syndrome decreased abdominal fat mass, blood pressure and hepatic steatosis [42], while the flesh also improved the blood lipid profile and attenuated the liver and kidney changes produced by the high-fat diet in obese rats [43]. Several local *Garcinia* fruits are also popular in Borneo; for instance, *G. parvifolia*, also known as “kandis” or Brunei cherry, is used for food, either consumed fresh or as pickles, and to treat sore throat and cough [44,45]. *G. forbesii*, also known as “aroi aroi”, is used for food as a cooking additive, for treating stomachache and for cough relief [44]; recent reports showed that *G. forbesii* has very good antioxidant properties [46]. *G. atroviridis*, locally known as “asam gelugur”, apart from being used in curries, has been used for treating itchiness and high blood pressure [44]. *G. dulcis*, also known as “mundu”, has an acidic taste and has several uses, including as an anti-pyretic, and the juice is used as an expectorant for cough and sore throat [47,48,49]. The fruit rind contains further bioactive phytochemicals including garcinol, morelloflavone and citric acid, which have beneficial metabolic effects in obese rats and can improve the gut microbiota [50]. Other reported biological activities include anti-inflammatory, hypolipidaemic, vasodilator, hypotensive and diuretic responses [51,52].

To efficiently extract the active ingredients from mangosteen, several methods have been employed, including a microwave-assisted method for α-mangostin extraction using ethyl acetate [53], anthocyanin isolation with an ultrasound technique using 50% ethanol [54] and another method, using liquefied dimethyl ether, was able to extract α-mangostin (42 mg/g dry sample) from 6 g of mangosteen peel powder [55]. Other sophisticated extraction methods also have been explored, including using a subcritical water state, allowing non-polar compounds extraction [56], and a supercritical water extraction method [57,58]. When comparing different solvents for the most suitable solvent for α-mangostin, high solubility of α-mangostin was shown in ethyl lactate, dimethyl carbonate, 2-methyltetrahydrofuran, ethyl acetate and ethanol [59]. Ethanolic concentrations of around 80% were most favourable for α-mangostin and γ-mangostin extraction [60]. The combination of slow freezing and hot-air drying was most suitable for α-mangostin extraction from the mangosteen rind [61]. The various extraction methods for α-mangostin have been reviewed, and the chosen method for α-mangostin extraction should be based on time, cost, sustainability and long-term environmental impact [62].

The use of inedible fruit parts as a source of phytochemicals and bioactive compounds is important to reduce environmental stress. Although α-mangostin is more abundant in the fruit rind of *G. mangostana*, anthocyanins such as cyanidin-3-sophoroside have been used as anti-browning agents in apple cuts [63] and as a natural colourant for jams [64]. The mangosteen fruit rind also has industrial applications such as using the carbonised mangosteen tissues, including the peel, for the removal of contaminants such as fabric dyes [65], metal ions and other wastewater contaminants [66,67]. The uses of activated carbon from mangosteen rinds are also being explored and several applications have been demonstrated, including use as a material to refine biodiesel and as a battery component [68,69]. Mangosteen has also been used as a modified carrier molecule: nano-fibrillated cellulose derived from its rind was employed to emulsify and encapsulate vitamin D, enhancing the compound’s bioavailability in the gastrointestinal tract [70]. Further industrial and functional applications of mangosteen have been discussed [67].

Based on our previous study using the obese rat model, we found that the equivalent dose of α-mangostin intake to an adult human was 1680 mg/day [42]. This translates to about 130 g of fresh mangosteen rind and approximately two fresh medium-sized mangosteen fruits. The use of this product would aid in using the whole fruits and reduce overall waste.

### 2.3. Rambutans and Pulasan

Rambutans (*Nephelium lappaceum*) (Figure 1), also known as the hairy litchi, is a fruit native to the Malaysian–Indonesian region including Borneo [71]. The colour of the peel ranges from deep red to yellow, with dense wavy hairs or soft spines [72]. The fruit flesh is white-translucent, juicy and tastes a bit acidic but mostly sweet [71]. The aril of the rambutans contains vitamins A, B_1_, B_2_, B_5_ and vitamin C and several minerals such as K, Mg and Na [71]. In Borneo, 16 different species of *Nephelium* have been reported [71]. A survey has shown that local communities in Sabah and Sarawak are aware of the usage of this fruit not only as food but also as medicine [44,73]. Rambutan peels have been reported to have several beneficial health effects such as anti-neoplastic, anti-microbial, hypoglycaemic, anti-aging, anti-allergic and anti-inflammatory properties [74,75,76,77,78]. Extraction methods have been developed to effectively extract anthocyanins from the rambutan peel, the compound associated with many positive health effects [75].

Another related fruit found in Borneo and Southeast Asia, *Nephelium mutabile*, synonym *Nephelium ramboutan-ake* (traditional name: “pulasan” or “kapulasan”) (Figure 1), closely resembling rambutan fruits, is also popular and gaining increased visibility [79]. This fruit contains active components such as tannin, phenolics, saponins and flavonoids [80]. The peel of pulasan from East Borneo has the highest phenolic content, which can be a promising source of phytochemicals [81]. The rind of the pulasan has been shown to have rich phytochemical contents including geraniin, catechin, chlorogenic acid, corilagin (an ellagitannin), syringic acid and naringenin, and the rind may function as anti-hypertensive and hypoglycaemic agents [82]. Additionally, the aqueous fraction of the pulasan rind has cytotoxic activity against HT-29 cells [80].

### 2.4. Langsat

Langsat (*Lansium domesticum*) (Figure 1) belongs to the Meliaceae family and is a popular tropical fruit with a sweet flesh but bitter seeds [83]. *L. domesticum* is a complex aggregate of species of different plant forms, which include “duku”, “dokong” (“longkong”) or “duku-langsat” and “langsat”, and slight morphological differences may exist [83]. The most common varieties are “duku” and “langsat”. The seeds of *Lansium* spp. are used as traditional medicine to treat various conditions such as an anti-pyretic agent [84], while the bark is also used as an anti-malarial agent [85] and the leaves are used as mosquito repellent [86,87]. The fruit also has potential as a cosmetic ingredient due to its moisturising and whitening effects [88]. The young langsat fruit has good antioxidant activities and anti-cancer properties [89], and the seed extracts also possess good antioxidant properties [90]. The fruit and plant parts of *L. domesticum* harbour a variety of compounds including onoceranoids, glycosides, cycloartanoids, tetranortriterpenoids, sesquiterpenoids and steroids [83], with the triterpenoid onoceranoid forming the main compound in this plant [91]. Although the fruit is popular due to its exotic taste, most traditional medicinal usages reported are based on other parts of the plant, including the seed, bark, wood, wood tar, resin and stem, with the reported uses in Borneo including moisturiser, talc powder, anti-fertility medicine and anti-pyretic [91].

### 2.5. Dabai

Another seasonal fruit that is native to Borneo is *Canarium odontophyllum* (Figure 1), locally known as “dabai” in Sarawak and Kalimantan and “kembayau” in Sabah and Brunei [92]. It is also known as “Borneo olive” or “Sarawak olive” [93,94]. The ripe fruit is dark purple or black, while the flesh is whitish yellow in colour. Due to the hard texture of the pulp, locals immerse the fruits in hot water for about 15 min to soften the flesh. The fruit is eaten locally to maintain health. There are many beneficial effects of this fruit; for instance, the fruit pulp has high contents of phenolics, flavonoids, anthocyanins and carotenoids [92,95]. Among the anthocyanins are malvidin glucoside, cyanidin glucoside, cyanidin rutinoside and peonidin glucoside [96]. The pulp has high antioxidant activities [95] and the seed has higher anti-cholinesterase activity than the pulp [92]. The dark dabai skin has the highest content of phenolics, flavonoids, free radical scavenging activity and β-carotene bleaching activity compared with the pulp and kernel [93], showing the potential of the skin as a source of natural antioxidants. The reported health effects of dabai fruit include its cholesterol-lowering effect in hypercholesterolaemic rats, which could be explained by the presence of fibre and phenolic compounds including 4-hydroxybenzoic acid, gallic acid and syringic acid [97,98]. The pulp is also increasingly extracted to produce oil, which has a comparable fatty acid profile to palm oil [99] and has hepatoprotective and cholesterol-reducing properties [100]. Additionally, the stem bark extract of dabai has cytotoxic effects against the HCT 116 human colorectal cancer cell line [94], while the leaf extract has anti-bacterial, anti-malarial, anti-diabetic and anti-hypertensive properties [101].

### 2.6. Native Mango in Borneo

*Mangifera indica* is the most well-known type of mango in Borneo, but there are other wild species that are endemic to this region and serve as exotic and medicinal sources of food. For instance, *Mangifera quadrifeda* (“asam kumbang”) is endemic to Brunei, Sabah, Sarawak and Indonesia, where they are made into pickles and sambals [102].

“Binjai” (*M. caesia*), commonly known as “belunu” in Sabah or “white mango” or “Borneo mango”, is a tropical wild fruit found in Borneo and throughout Southeast Asia [44,103]. It belongs to the same genus as the mango (*M. indica*) and is characterised by its strong aroma, creamy white flesh and sweet–sour taste. Indigenous to the rainforests of Borneo, binjai is popular in local markets and is often enjoyed fresh. Apart from its delicious taste, binjai holds a cultural and medicinal value for the communities in Borneo. Traditionally, it has been used in local medicine and is known for its various health-promoting properties. For example, it is used to increase appetite and treat colds, itchiness, high blood pressure and bronchitis [44]. Its unique nutritional profile makes it a valuable part of the local diet and an interesting fruit for those looking to explore functional foods from Southeast Asia. About 65% of the binjai fruit is edible. Apart from macronutrients, the fruit also contains thiamine, β-carotene and vitamin C [103]. A comparison with other native mangoes in Malaysia showed that it has the highest antioxidant activities, as evidenced by its high free radical scavenging activity and high total flavonoid content [104].

Perhaps the most well-known mango indigenous to Borneo is *M. pajang* (Figure 1). Locally known as “bambangan”, “embang” or “mawang”, the fruit is popularly used in local Bornean dishes [105]. The edible pulp forms 60–65% of the total weight and has a bright yellow colour, is fibrous and has a characteristic sweet–sour taste [106]. The pulp can be eaten fresh but is regularly fermented to give a longer shelf life, and it forms an essential part of the traditional Kadazandusun cuisine [107]. The aromatic peel is also used to provide acidic taste in curries [105]. The pulp contains a considerable amount of ascorbic acid, naringenin and hesperidin in addition to phenolic acids, flavonoids, carotenoids and xanthophylls [105,108]. The peel of *M. pajang* is abundant in beneficial phytochemicals including flavonoids (catechin, daidzein, epicatechin, hesperidin, kaempferol, naringin, luteolin, quercetin) and phenolic acids (gallic acid and *p*-coumaric acid, chlorogenic acid, ellagic acid, ferulic acid, gallic acid, 4-hydroxybenzoic acid, protocatechuic acid, vanillic acid and pyrogallic acid) [108]. The unique compound mangiferin is also found in the peel of *M. pajang* in higher concentrations than in the peel of *M. indica* [109,110]. The kernel of *M. pajang* is also rich in phenolic acids and flavonoids, where the major components are ferulic acid and diosmin [108,111]. The peel and seeds of this fruit have the highest amounts of mangiferin, supporting the use of these fruit parts as a good source of nutraceuticals [112]. The fermented *M. pajang* fruits possess good antioxidant activities (measured through ferric reducing antioxidant power assay and trolox equivalent antioxidant capacity assay) and total phenolic acid content, with the major phenolic compounds identified as gallic acid, chlorogenic acid, vanillin, *p*-coumaric acid and rutin reported to be good antioxidants [113].

An increasing number of studies have reported the medicinal effects of *M. pajang*; apart from its well-reported antioxidant properties, particularly from the kernel [106], methyl gallate from the fruit was shown to have a strong anti-bacterial activity against methicillin-resistant *Staphylococcus aureus* [114]. Additionally, the kernel extracts of *M. pajang* have promising fungistatic or fungicidal activity against human pathogens [115]. Several *in vitro* anti-cancer studies have been conducted and the ethanolic extract of the kernel and peel showed cytotoxicity against ovarian, colon and liver cell lines [108]. The kernel crude extract also showed cytotoxicity by apoptosis in hormone-dependent breast cancer cells (MCF-7), through cell cycle arrest at sub-G1, and non-hormone-dependent breast cancer cells (MDA-MB-231), through G2/M arrest [116]. Methyl gallate isolated from the methanolic extract of the *M. pajang* kernel demonstrated induction of apoptosis in human breast cancer cells (MCF-7) through oxidative stress mechanisms [117]. In humans, the consumption of *M. pajang* juice powder for 9 weeks increased the plasma β-carotene and ascorbic acid concentration and plasma total antioxidant status in healthy subjects [118]. When tested for its *in vitro* antioxidant activities, *M. pajang* juice showed high diphenyl-1-picrylhydrazyl radical scavenging activity, high total phenolic content and high inhibition of malondialdehyde formation [119].

### 2.7. Artocarpus Species

*Artocarpus odoratissimus* (Figure 1), locally known as “tarap” in Sabah and Brunei and “marang” in the Philippines, is a popular tropical fruit indigenous to Southeast Asia [106,120]. Belonging to the *Artocarpus* genus alongside jackfruit, cempedak and breadfruit, this seasonal fruit uniquely combines an alluring aroma, velvety texture and succulent flesh. Widely cultivated in Sabah and Sarawak in Malaysian Borneo as well as in parts of the Philippines and Indonesia, tarap is a favourite among locals. Tarap fruit has a distinctive appearance characterised by a rough, spiky green shell when unripe that turns yellowish-brown as it matures. The spikes are soft and flexible compared with other spiky fruits such as durian. The fruit is oval to oblong in shape and can range in size from about 15 to 25 cm in length. When opened, the tarap reveals clusters of creamy, bulbous arils surrounding a central core. Each aril is soft and juicy, encasing a seed inside. The flesh of the arils is creamy-white to pale yellow and exudes a fragrant aroma that is both sweet and slightly musky. The seeds within are whitish brown and round or oblong. The seeds are often roasted and consumed as a healthy snack, adding a source of protein and fibre to the diet. The fruit has recently attracted scientific interest due to its nutrient-rich profile and potential health benefits, which include antioxidant and anti-inflammatory properties. Beyond its culinary appeal, tarap is rich in essential minerals, such as K, Fe and Zn, and various vitamins [121,122]. The tarap fruit has anti-bacterial, anti-cancer and anti-diabetic properties [123], while the leaves of this plant are used for treating gout [124].

Another popular fruit in Borneo from the jackfruit family is cempedak (*Artocarpus integer*) (Figure 1). This unique fruit is celebrated for its sweet, aromatic flavour and distinctive, soft texture. In Borneo, cempedak is often enjoyed both fresh and cooked, with the young, unripe fruit being a popular ingredient in savoury dishes, while the ripe fruit is typically consumed fresh or fried [125]. It is high in dietary fibre, which aids in digestion, helps maintain bowel health and promotes satiety. Vitamin C is abundant in cempedak, contributing to immune health, skin integrity and antioxidant defence, as are the B group vitamins. Cempedak is also rich in minerals such as Ca, Mg and Fe as well as phenolic compounds [126]. Breadfruit, scientifically known as *A. altilis* and locally known as “sukun”, is a tropical fruit native to the Indo-Malay region and is widely cultivated in Borneo and other parts of Southeast Asia [127]. This fruit is cherished for its unique texture and flavour, which resembles freshly baked bread when cooked. Breadfruit is commonly consumed in various forms, both ripe and unripe, but it is particularly enjoyed when cooked. Like tarap and cempedak, sukun is not only delicious but also packed with essential nutrients that contribute to health, such as carbohydrates, mainly in the form of starch, dietary fibre, amino acids and minerals in addition to beneficial phytochemicals [128]. *A. sarawakensis*, also known as “pingan” in Sarawak, is another fruit that resembles the well-known *A. odoratissimus*, and the fresh fruit is usually eaten [129]. However, reports on its biological activity are very rare.

In Borneo and across Malaysia, young or unripe “nangka” (jackfruit, Figure 1), *Artocarpus heterophyllus*, is a popular ingredient in traditional cuisine. Unlike the sweet, ripened jackfruit, the young fruit is mildly flavoured with a tender texture that readily absorbs spices and seasonings, making it ideal for savoury dishes [125,130]. In Borneo and other parts of Malaysia, young jackfruit is often prepared in curries such as “gulai nangka”, where it is slow-cooked with coconut milk, turmeric, garlic and other spices to achieve a fragrant and flavourful dish. It is also used in soups and stir-fries combined with local ingredients such as chillies, lemongrass and “daun salam” (Indonesian bay leaf), contributing to the rich culinary heritage of the region [131]. The Dayak Jangkang tribe from West Kalimantan also shared this culinary practice, showing the similarities of usage within the island [125].

Nutritionally, young jackfruit is dense with essential nutrients that contribute to a balanced diet, offering carbohydrates, dietary fibre and vitamins C and B_6_. It is also a source of minerals such as Mg, Ca and Fe, which support heart health, bone density and overall vitality [131]. Additionally, young jackfruit contains phytonutrients, including phenolic compounds and flavonoids, that have antioxidant properties beneficial for protecting cells from oxidative stress and reducing inflammation in the body [130,131]. These bioactive compounds enhance the fruit’s role in traditional diets, where it is valued for both its culinary uses and potential health benefits.

### 2.8. Baccaurea Species

*Baccaurea lanceolata* (Figure 1), locally known as “liposu” in Sabah and “limpasu” in Kalimantan, Indonesia, is another indigenous fruit thriving in the diverse ecosystems of Borneo, the Philippines and Thailand. Characterised by its small, round shape and translucent white flesh with a sour yet refreshing taste, this fruit contains important minerals [132]. Enjoyed year-round, it is commonly consumed either by dipping into sugar or salt, as fruit juice, incorporated into cooking as fruit slices or made into sambals [133,134]. In Indonesia, it also serves as a remedy for acne and a natural sunscreen [133,135,136]. Traditionally, it has been used to address various ailments, such as stomachaches, headaches and diarrhoea and to alleviate drunkenness [133,137,138]. The phytochemical contents and antioxidant activity of *B. lanceolata* highlights the strong correlation between the phenolic compounds and antioxidant activity, particularly in the flesh [139]. In addition, studies on different parts of the tree and fruits have explored anti-microbial activity against various bacterial strains, revealing medium to potent inhibition [138,140].

### 2.9. Averrhoa Species

“Belimbing buluh” or “belimbing besi” (*Averrhoa bilimbi*) (Figure 1), known as “tulod ulod” by the indigenous people in Sabah, is a tropical fruit native to Southeast Asia and widely cultivated as a garden tree [141,142]. It is lesser known than *A. carambola* or starfruit [33]. In Borneo, belimbing buluh is commonly used as a souring agent in various dishes, adding a tangy flavour to dishes. It is often included in traditional salads, where it is mixed with other vegetables and served with spicy sauces. The fruit can also be pickled or as “sambal” (chilli paste), providing a delicious accompaniment to meals. Additionally, belimbing buluh is sometimes consumed raw, providing a refreshing and tart snack. Belimbing buluh’s fruit extracts contain carbohydrates, amino acids, flavonoids, tannins, flavonoids, bitter constituents, essential oils, coumarin, terpenes and valepotriates. The fruits also contain considerable amounts of vitamin C and oxalic acid [142]. The fruit is appreciated for its medicinal properties and is used in traditional remedies throughout the region, for example, as a treatment for cough, fever, inflammation, skin conditions and diarrhoea and as a diuretic [143].

### 2.10. Wild Berries

Wild berries (*Rubus* spp.) in Borneo have been traditionally used as a source of additional diet and medicine in local communities. There are several species that are native to Borneo; for instance, *R. rosifolius* (roseleaf raspberry, Figure 1) is a species native to Sabah and Sarawak [144]. In Sabah, several species have been reported and studied, including *R. moluccanus*, *R. fraxinifolius* and *R. alpestris*, where they are collectively known as “rogimot” among native Kadazandusun tribes [145,146]. In Sarawak, *R. glomeratus* (emperingat), *R. alpestris* (emperingat), and *R. moluccanus* (akar) have been reported in Lanjak Entimau Wildlife Sanctuary [147]. *R. fraxinifolius* also thrives in Kalimantan, Indonesia [148]. These berries are rich in phenolic compounds, carotenoids, flavonoids and anthocyanins [145,146]. These berries have antioxidant, anti-inflammatory and anti-bacterial properties and inhibit acetylcholinesterase activity [145,149]. The medicinal uses reported of the *Rubus* species include medicine for diarrhoea, dysentery, fungal infections, cough and headache [147,149]. However, biological studies are still limited and need further investigation.

### 2.11. Pangium Species

“Buah kepayang” or “buah keluak” (*Pangium edule*, Figure 1), or “pangi” as it is known by the indigenous people in Sabah, is a wild-growing plant found across many Southeast Asian countries [150,151]. Most parts of the plant, including its leaves, bark and seeds, contain cyanogenic glycosides, making them naturally toxic [152]. When ripe, the aril has a mildly sweet taste, but the seeds are the most valued part of the plant. The seeds of pangi are high in fat and harbour gynocardin, a glycoside that releases hydrocyanic acid during the ripening process [152]. Despite their natural toxicity, pangi seeds are edible after undergoing specific treatments, such as soaking and steaming [153]. In Indonesia, fermented seeds are a culinary specialty and are used as a spice and preservative agent [154]. In the northern regions of Borneo (East Malaysia), dried pangi seeds are ground into powder and incorporated into traditional dishes and indigenous fermented foods. It is believed that the addition of these seeds helps regulate the fermentation process and imparts a unique flavour to the products. Additionally, the plant has traditional medicinal uses, such as for wound healing, and has been employed as a fishing poison [152]. Scientific studies have reported several biological effects of this fruit including anti-microbial, antiviral, anti-fungal and anti-cancer properties [150,152,153,155].

### 2.12. Other Underutilised Fruits

“Pengolaban” (*Litsea garciae*, Figure 1) also known as “engkala” or “engkalak”, is an endemic fruit that is less known in Sabah. This fruit has high anthocyanin and antioxidant contents, which are beneficial for health [156]. *Baccaurea angulata*, also known as belimbing dayak or belimbing hutan, is widely available in Borneo and other regions of Indonesia. The fruit flesh is eaten fresh and the skin is used in cooking. The fruit contains anthocyanins, phenolic compounds and flavonoids, which account for its high antioxidant activity [157].

“Engkabang” fruit (*Shorea macrophylla*) is the largest genus of the *Dipterocarpaceae* family and is a popular local fruit found in tropical forests or riversides in West Kalimantan (Indonesia) and Sarawak (Malaysia). This fruit is also known as illipe nuts, “kawang” in Brunei and “tengkawang” in Indonesia [158]. It produces a good amount of oil and has been used as a source for oil in feed products, cooking oil, cosmetics and other edible products [158]. The production of engkabang oil is seasonal, and the butter oil is usually found in local markets in Sarawak towns such as Sibu, Miri and Kapit [159]. The main constituents of the oil are stearic acid, oleic acid and palmitic acid, with arachidic acid and linoleic acid also present in smaller quantities [160]. Despite its popularity, more research still needs to be performed on its phytochemical constituents and health properties.

Snakefruit (*Salacca* spp., Figure 1) is also widely available in Borneo. In Sarawak, for example, *S. affinis* is used as a cooking ingredient mixed with chilli and shrimp paste, while *S. magnifica* is eaten raw [32]. In Sabah, four varieties of *S. zalacca* have been cultivated that show high phytochemical and antioxidant properties [161] and have anti-inflammatory, antioxidant and anti-diabetic activities [162,163]. The reported phytochemicals in *Salacca* spp. include flavonoids, alkaloids, terpenoids, tannins, sitosterols, quinones and carboxylic acids [163].

“Empelanjau” or “pelajau” (*Pentaspadon motleyi*) has ovoid fruits that are sharply pointed. The fibrous husk covering the small kernel is discarded to recover the white kernels. The white kernels are nutty in flavour and are boiled or cooked with other vegetables or fried for making snacks [164]. “Kubal tabau” (*Willughbeia sarawakensis*) bears pear-shaped fruits weighing 1 kg or more. “Kubal madu” (*W. augustifolia*) is reported to have a soft aril and has an acidic to sweet taste. It is usually available in Tamu market when in season and has big potential for domestication and commercialisation [164].

Some other underutilised fruits in Malaysia have been reported previously [165]. Fruits belonging to the *Baccaurea*, *Durio*, *Garcinia* and *Mangifera* genera are mostly reported to be understudied for their phytochemical properties, nutritional composition, antioxidant capacity and therapeutic properties [165]. Hence, plans to increase their presence among the public and to enhance their culinary and functional applications should be implemented by the relevant bodies.

## 3. Fermented Foods

Fermented foods are foods and beverages that are made by using specific microbial-based fermentation aids such as yeast and bacteria [166]. Through controlled enzymatic processes, these microorganisms transform food components into useful products for healthy fermentative activities. Fermentation also aids in preserving food products and inhibiting the growth of pathogenic microorganisms [166]. Fermented foods have long been an integral part of culinary traditions worldwide, serving as both a preservation method and a means of enhancing flavour, texture and nutritional content. In Borneo, plant-based fermented foods are a cornerstone of the diverse culinary heritage. These traditional practices have been shaped by the island’s rich biodiversity, cultural diversity and reliance on local ingredients. The fermented foods in Borneo are usually made by the local indigenous people to preserve fruits and vegetables to provide longer shelf time [167]. The fermented product is usually eaten with rice as an appetiser [105].

**Figure 2:**
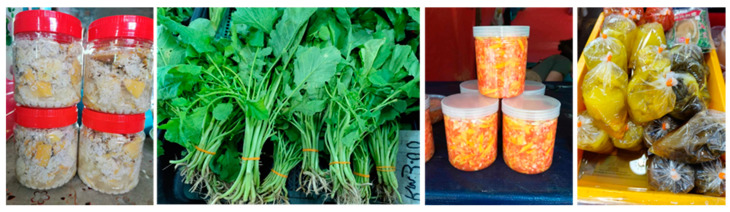
Fermented plant-based foods from Borneo. From left to right—fermented bambangan (*Mangifera pajang*), sarawak ensabi (*Brassica juncea*) used to make the kasam ensabi, tuhau pickles made from wild ginger (*Etlingera coccinea*) stalks, assorted fermented foods sold in a local market in Miri, Sarawak.

Borneo’s traditional fermented foods include a variety of plant-based products such as “tempoyak”, “kasam ensabi”, “tuhau pickles” and “tapai/lihing”, among others [168,169,170]. These foods are typically produced using indigenous vegetables, salt and a natural fermentation process. Tempoyak is one of the most well-known dishes that is made from ripe durian; this food is added with salt and fried together with other foods or used as paste seasoning and has great potential to be used as a probiotic food [168]. The fermented fruit of bambangan (*M. pajang*, Figure 2) is also very popular in Sabah. The fermentation is achieved by mixing the ripe bambangan flesh with its grated seed and salt and left for fermentation in a closed container at ambient temperature for 7–10 days [171]. The most common microorganisms isolated from the food product are *Lactiplantibacillus plantarum* and *Pediococcus pentosaceus*, while *Candida krusie* and *Kloeckera apis* become the major organisms in the later fermentation process.

One notable example of fermented food from Sarawak is “kasam ensabi” (Figure 2), a fermented mustard green (*Brassica juncea*) commonly prepared by the Iban community in Sarawak. The process involves salting the mustard greens, pressing them to remove moisture and storing them in airtight containers to ferment for weeks [172]. The fermentation not only preserves the vegetables but also imparts tangy, umami-rich flavours that complement rice-based meals.

Another popular fermented vegetable-based food in Borneo is the “tuhau” pickles made from stalks of wild ginger (*Etlingera coccinea*) (Figure 2), a plant native to the region. The stalks are finely chopped, mixed with salt, and added with either vinegar or lime juice and chillies to create spicy, aromatic pickles. This dish is especially cherished by the Kadazandusun community in Sabah, who value its distinctive flavour and its role as a condiment in everyday meals [173]. The “pangium” seeds are popular in north Borneo as fermentation ingredients as they produce a unique taste and texture after fermentation with meat or fish [174]. Another local fermented food among the local Kadazandusun ethnic group in Sabah is called “bosou”; although mostly made from meat or fish, vegetables bosou is also widely available for consumption [175]. Other popular fermented foods in Sarawak include fermented dabai and fermented cassava leaves [167]. These food products pose a great opportunity for future probiotics study [167].

Fermentation enhances the nutritional profile of these foods, making them a valuable component of the Bornean diet. The microbial activity during the fermentation process increases the bioavailability of nutrients, synthesises nutrients such as B group vitamins and reduces anti-nutritional factors such as phytates [176,177]. For instance, the fermented mustard greens in “kasam ensabi” are rich in probiotics, which support gut health and improve digestion [167]. Similarly, the wild ginger in tuhau pickles is known for its antioxidant and anti-microbial properties, which are enhanced during fermentation [178].

Studies on the microbial diversity of fermented foods in Borneo reveal a dominance of lactic acid bacteria such as *Lactobacillus* and *Leuconostoc* species [167]. These microbes play a crucial role in lowering pH, inhibiting spoilage microorganisms and contributing to the unique flavours and textures of the fermented products. The consumption of lactic acid bacteria-rich foods is linked to numerous health benefits, including immune modulation, reduced inflammation and improved gastrointestinal health. In the three traditional foods from Kalimantan, which are cincalok, tempoyak and mandai, five lactic acid bacteria were identified; cincalok was dominated by *Tetragenococcus*, whereas both tempoyak and mandai have more *Lactobacillus*, indicating that these foods have great potential as probiotic sources [179].

Vegetable-based fermented foods hold immense cultural value in Borneo, serving as symbols of heritage and identity. Traditional fermentation techniques are often passed down through generations, fostering a sense of continuity and community. In many indigenous groups, these foods are prepared collectively during harvest seasons or festive occasions, reinforcing social bonds. In addition to their domestic use, fermented foods are integral to rituals and ceremonies. For instance, kasam products are often served during weddings, festivals and communal gatherings, signifying abundance and hospitality (Figure 2). The production of fermented foods also reflects the resourcefulness of Borneo’s communities, who use fermentation to extend the shelf life of seasonal produce, ensuring food security during lean periods [180].

Despite their importance, traditional fermented foods in Borneo face challenges due to urbanisation and modernisation. Younger generations are increasingly drawn to convenience foods, leading to a decline in traditional practices. Moreover, the lack of standardised fermentation processes can result in inconsistent quality and potential safety issues, such as microbial contamination. Nonetheless, the global interest in natural and functional foods has sparked initiatives to revive and commercialise these traditional products. Efforts to document recipes, conduct scientific studies and develop safer fermentation techniques are underway. For instance, research on microbial profiling and nutritional benefits aims to bridge the gap between traditional knowledge and modern food science.

Borneo’s unique biodiversity significantly influences its fermentation practices. The island is home to numerous endemic plant species used in traditional fermented foods. Indigenous vegetables such as wild ferns, bamboo shoots and tropical greens are often fermented using natural starters derived from the local environment, showcasing a deep connection between communities and their ecosystems.

For example, bamboo shoot fermentation is prevalent among the Dayak communities in Kalimantan. Fresh shoots are sliced, salted and fermented in bamboo containers to produce “sayur masam”, a sour and crunchy dish [181]. This process exemplifies sustainable food preservation methods and highlights the ecological knowledge of indigenous groups. Compared with other regions, Borneo’s vegetable-based fermented foods are notable for their reliance on tropical ingredients and simple fermentation techniques. Unlike the more elaborate processes in other East Asian cuisines, Borneo’s methods emphasise natural processes and locally available resources, showcasing the adaptability and ingenuity of its communities.

Future research on Borneo’s fermented foods holds great potential. Areas such as microbial profiling, nutritional analysis and the development of novel fermentation starters could improve product quality and promote health benefits. Incorporating traditional fermented foods into modern diets through innovative recipes and packaging could attract younger consumers and international markets.

## 4. Borneo Seaweeds

Traditionally, seaweed cultivation has served as a supplementary source of income for coastal communities, where fishing is the primary economic activity for most residents. On the east coast of Sabah such as Semporna, Kunak, Tawau and Lahad Datu, as well as on Banggi Island in northern Sabah, the marine environment is favourable for farming *Eucheuma* and *Kappaphycus* species, primarily for producing carrageenan [182].

For centuries, seaweed has been traditionally used as food in China, Japan and Korea. As people from these countries migrated globally, they brought this culinary tradition, making seaweed consumption increasingly common in many other parts of the world, including the coastal communities in Borneo, particularly in Sabah. Some of the common species of seaweeds found in North Borneo are *Kappaphycus alvarezii*, *Eucheuma denticulatum*, *Halymenia durvillaei*, *Caulerpa lentillifera*, *Caulerpa racemosa*, *Dictyota dichotoma* and *Sargassum polycystum* [183,184]. A comprehensive review of their therapeutic potential has been published previously [183]. Among these edible seaweeds, “latok”, or sea grapes (*Caulerpa* sp.), are the most widely consumed as salad in the diet of the coastal communities in Sabah; they have been reviewed for nutrient composition and health benefits [183,185]. Nutrients from sea grapes include carbohydrates and fibre, proteins and amino acids, lipids and fatty acids, minerals, vitamins, pigments and antioxidant components [185]. Meanwhile, the health benefits include cardioprotective (i.e., anti-hypertensive and hypolipidaemic), anti-bacterial, anti-cancer, anti-coagulant, anti-diabetic, anti-inflammatory, antioxidative, anti-pyretic and immunostimulatory properties as well as chelating agent activity [183]. In addition to sea grapes, Sabahans also consume dried *K. alvarezii* and *E. denticulatum* as salad foods, which have been studied for their nutrient contents and health benefits [186,187].

**Figure 3:**
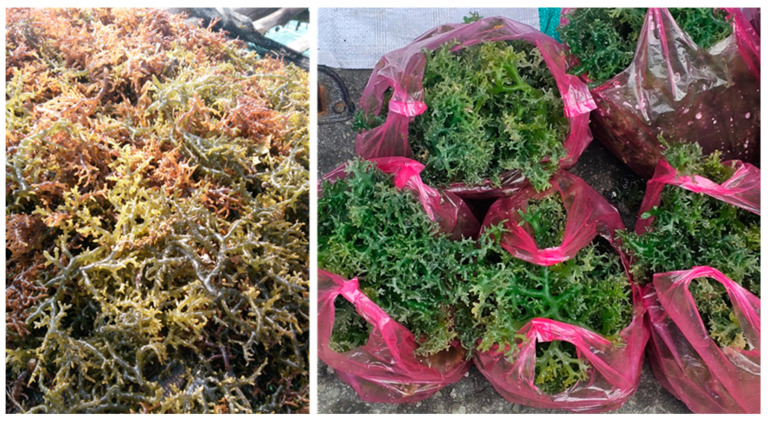
*Kappaphycus alvarezii* from Borneo: (**left**) fresh from the sea; (**right**) as sold in a local market.

The terms “macroalgae” and the more colloquial “seaweeds” generally refer to multicellular marine photosynthetic organisms with a plant-like holdfast living in shallow coastal areas [188]. The number of known species has been reported as 2033 brown, 7083 red and 1901 green macroalgae, a total of 11,017 species [189]. New species are likely to be found in tropical coastal marine systems such as coral reefs and mangrove forests, common in the seas surrounding Borneo [190]. Global production of macroalgae increased threefold between 2000 and 2019, with China producing over 56% of the world production, Indonesia the second largest producer with over 28%, the Philippines producing over 4% and Malaysia producing 1.5% [191]. The most common species in Indonesia and Malaysia are *Eucheuma* spp., *Gracilaria* spp. and *K. alvarezii* (Figure 3). In Asia, 99% of macroalgae production is artificially farmed. This farming technique provides the expertise to decrease future resource crises using tropical seaweeds as a resource leading to environmentally sustainable and socially equitable outcomes [192,193]. Nutrition products, ethanol and biogas are the most common outputs from macroalgal biomass, with lifecycle analysis used to improve large-scale commercial production [194]. Challenges of commercial seaweed production include biosecurity risks such as viral and bacterial diseases, aquaculture pests, epiphytes attached to seaweed surfaces and herbivory grazing; cost-saving mitigation methods include integrated multi-trophic aquaculture [195].

Macroalgae are a versatile health-promoting product, for example, to prevent or reverse the hallmarks of ageing by their actions on chronic inflammation and gut dysbiosis [196]. Increased accessibility to macroalgae requires increasing the range of species available for human consumption [196]. Macroalgae from Borneo may be a partial solution as they have potential therapeutic effects including protection of the heart, blood vessels, kidney, liver and nervous system, especially with antioxidant and anti-inflammatory actions [183]. The authors suggest that only *K. alvarezii* and *E. denticulatum*, mostly cultivated for carrageenan extraction, and *Caulerpa lentillifera* and *C. racemosa* are now consumed as food in North Borneo. Commercialisation to lead to more extensive use as therapeutic agents requires the determination of the concentrations of potential therapeutic compounds and their biological activity as well as of issues such as the loss of active compounds on storage or cooking, bioavailability and the potential accumulation of toxic compounds.

The phytochemicals found in Indonesian seaweeds that were studied in preclinical studies, usually on isolated enzymes that modify blood glucose concentrations or in rodents with chemically-induced diabetes, have indicated promising leads for the future development of anti-diabetic drugs [197]. However, there do not seem to be clinical reports of the translation of these results to diabetic humans, especially in Borneo. Cardiovascular protective effects such as reduced obesity and improved blood lipid profiles have been shown after administration of *K. alvarezii*, *Caulerpa lentillifera* and *S. polycystum* grown in Sabah in rats fed a diet high in cholesterol and fat [198]. Rats given a high-fructose and high-saturated-fat diet produce similar physiological changes to metabolic syndrome in humans; in these rats, tropical seaweeds including *K. alvarezii* [199], *Sarconema filiforme* [200], *C. lentillifera* [201] and *Sargassum siliquosum* [202] improved cardiovascular and liver structure and function and altered the gut microbiome. The addition of macroalgae to human food, especially the macroalgal polysaccharides, lipids and proteins, can improve both the nutritional and textural qualities of the diet and improve consumer acceptability [203]. Further, human health may be improved by the indirect effects of increased production of macroalgae, including wastewater remediation, carbon capture, production of bioenergy and biofertilisers, and decreased methane production by ruminants. To achieve this outcome, an improved understanding of the molecular and physiological processes in macroalgae is essential [13].

In addition to local food customs, research projects have studied the inclusion of seaweeds in recipes and food products with improved acceptability [203]. In Sabah, seaweed is popularly consumed as a salad and it is easily available in local weekly markets. In addition, seaweed can be blended with different fruits and vegetables in soups or salads [204]. *K. alvarezii* puree also has been made into fish sausages, flat rice noodles, yellow alkaline noodles, soy crisps and bakery products, which have promising potential to be included as functional foods [205,206,207]. The seaweed biomass from Sabah has been brought over to Peninsular Malaysia to be made into various seaweed products such as health drinks, desserts, soaps and air fresheners [208].

An important issue to be considered when consuming seafood and seaweed from Borneo is the seasonal occurrence of “red tides” or toxic algal blooms [209]. Several cases have been reported due to paralytic shellfish poisoning caused by *Pyrodinium bahamense*, a dinoflagellate producing saxitoxin. Other cases including *Cochlodinium polykrikoides*, *Gymnodinium catenatum*, *Gonyaulax polygramma* and *Noctiluca scintillans* have been reported. Consistent monitoring has reduced toxicity cases due to the timely warning given by the Department of Fisheries; consumers must be aware of the current situation of red tide before consuming seafood in the affected areas [209]. Further, as seaweed contains a variety of polysaccharides and proteins that contain various chemical groups such as anionic carboxyl, sulphate and phosphate groups, they have a high affinity for heavy metals, and hence, they could accumulate and become a source of As, Cd, Pb, Hg and other compounds [203]. Seaweeds in Hainan Island, South China Sea showed that the accumulation of heavy metals varies according to seaweed types (red algae—V, Se, Mn, Ni and Ag; green algae—Zn and Pb; brown algae—Cr, Co, Cu, Cd, As and Fe). The Hazard Index was greater than 1 for seaweeds such as *Turbinaria ornate*, *Sargassum oligocystum*, *S. polycystum* and *S. thunbergia* when factoring the consumption by children, hence suggesting that the intake of these seaweeds should be controlled in children [210].

Microalgae are microscopic single-celled organisms living in aquatic environments, with green microalgae having led to all terrestrial plants [211]. Suitable local microalgae for biodiesel production have been identified in Sabah [212], while industrial cultivation of microalgae in Borneo for biofuels has now been achieved [213]. Microalgal biomass contains essential lipids, proteins, carbohydrates, vitamins and minerals, providing a useful alternative for the alleviation of global undernutrition [214]. Although microalgae are accepted as important and viable future foods [215], no reports were found on the use of microalgae in the diets in Borneo, but there is potential for the extraction of bioactive phytochemicals and food components, and for industrial uses, from these organisms, as reported in Sarawak [213].

## 5. Vegetables

Vegetables are an important source of essential vitamins, minerals, fibre and phytochemicals essential for human health [29]. Wild vegetables from Borneo hold a rich heritage of nutrition, biodiversity and cultural significance. As one of the most ecologically diverse regions in the world, Borneo offers a unique array of wild edible plants, many of which are deeply woven into the diets and traditions of the indigenous communities [141,216,217,218]. These vegetables are often foraged from the forest or grown in small community gardens, making them sustainable and accessible food sources. These vegetables are valued by local communities for promoting heart health, aiding digestion and providing anti-inflammatory properties due to their content of vitamins, minerals and antioxidants [219]. The wild vegetables of Borneo have potential beyond the island for their nutritional qualities and potential as sustainable food sources. Exploring the value of these vegetables sheds light on the importance of preserving Borneo’s unique biodiversity and indigenous food knowledge.

Vegetables form an integral part of the local Bornean diet, which includes wild mushrooms (oyster and tree-ear mushrooms); edible ferns, also known as “paku”, with a few varieties such as “paku ikan”, “midin” and “nyeral”; hairy eggplant, also known as “terong bulu”, and sour brinjals (eggplants) or “terong assam” or “terong dayak”. Further vegetables include stinky beans or “petai” and ginger blooms (*Etlingera elatior*) or “bunga kantan”, as well as stems such as *Etlingera coccinea*, also known as “tuhau”, or shoots such as bamboo shoots, also known as “rebung” [220]. These vegetables are in addition to the other commercially available vegetables such as cabbages, bok choys, beans, kales, spinach and other varieties including gourds and melons used as vegetables. The different varieties of vegetables in Borneo have been discussed previously [219]; hence, this section discusses the main vegetables that are widely used in Borneo.

### 5.1. Terung Dayak

**Figure 4:**
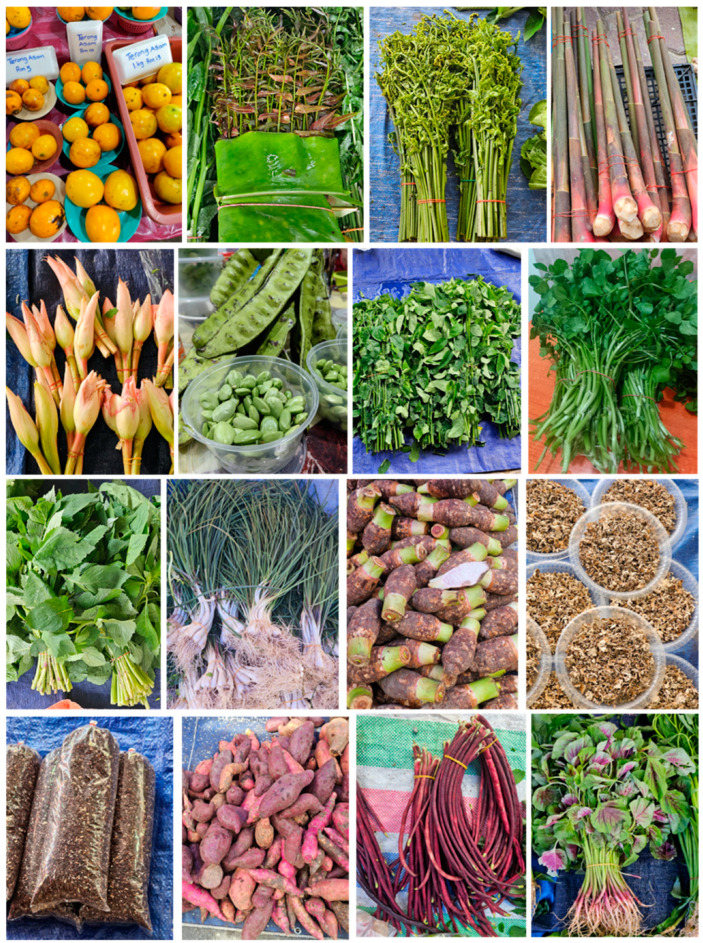
Vegetables from Borneo. First row—(left to right) terung dayak (*Solanum lasiocarpum*), lemiding/midin (*Stenochlaena palustris*), pakis (*Diplazium esculentum*), wild ginger (*Etlingera coccinea*); second row—(left to right) torch ginger (*Etlingera elatior*), petai (*Parkia speciosa*), sayur manis (*Sauropus androgynus*), wild watercress (*Nasturtium officinale*); third row—(left to right) tanduk manggarang/gipun (*Crassocephalum crepidioides*), chives/losun (*Allium* sp.), keladi (*Colocasia* spp.), kulat kodop (*Schizophyllum commune*); fourth row—(left to right) tadong rice (*Oryza sativa* var. Tadong), sweet potatoes (*Ipomoea batatas*), purple long bean (*Vigna unguiculata*), bayam merah/red spinach (*Blitum rubrum*).

The “terung dayak”, *Solanum lasiocarpum* (Figure 4), previously *S. ferox*, is also known as “terung asam” or sour eggplant and is native to Borneo, especially in the Sarawak region. This underutilised crop possesses a distinct sour and tangy flavour profile, making it a prized ingredient in local cuisine [221]. Morphologically, terung dayak is characterised by its spiny exterior and elongated, often curved fruit, which turns from green to yellow-orange when mature. The plant’s adaptability to diverse climatic conditions and its resilience to pests and diseases make it a valuable resource for local communities [222]. This fruit vegetable is popularly used in fish curry dishes, pickled or fermented [223]. Recently, terung dayak has been used for making cakes, biscuits and jams, and as dehydrated slices or pickled in brine [222]. Beyond its culinary significance, terung dayak has garnered attention for its potential health benefits. Its traditional uses include for treating allergies, body swelling and aches, skin injuries, headaches and as an anti-microbial, anti-cariogenic and anthelmintic treatment [224]. Preliminary studies suggest that it contains various bioactive compounds, including antioxidants such as vitamin C and polyphenols, which may contribute to the prevention of chronic diseases [224]. Further research is needed to evaluate the phytochemical composition and pharmacological properties of this indigenous vegetable.

### 5.2. Ferns

The most common vegetables in Borneo are perhaps of the leafy varieties, which are mostly eaten as salad or “ulam” [219]. Fern species are eaten as traditional vegetables in different parts of Borneo, although there are differences in ethnic diversity including the Kadazandusuns in Sabah, Dayak in East Kalimantan, Ibans in Sarawak and Dusun Merimbun in Brunei [219]. *Stenochlaena palustris* is a popular fern variety in Borneo (Figure 4), known as “lemiding”, “midin”, “kalakai” or “pakis merah” and commonly eaten raw, blanched or fried with garlic, anchovies and shrimp paste [219]. Its traditional uses include to increase breast milk production [225] and to treat infected wounds [226]. The leaf ethanolic extract contains the highest content of flavonoids and phenolics and exhibits the highest antioxidant activities compared with the other plant parts [227]. The mature fronds also contain hydroxycinnamic acids and show high antioxidant activity, while the young fronds contain more anthocyanins and show metal-chelating activity [228]. Further, the extract of *S. palustris* has anti-plasmodial activity [226,229]. The biological and health properties of *S. palustris* show the potential range of benefits of this exotic vegetable [225].

Another fern, *Diplazium esculentum* (Figure 4), is a popular edible plant in Borneo [230] known as “pakis” (Sabah), “paku ikan” (Sarawak) or “bajey” (Kalimantan) [219,231]. The plant is widely used in Southeast Asia, where the leaves and rhizome are boiled and used as an herbal drink in Indonesia for dysentery, to treat acne, tumours and asthma [232,233,234]. Phytochemical analysis showed that the plant has various metabolites including polyphenols, tannins, flavonoids, saponins, alkaloids and steroids [235]. Another study screening the phytochemical content of *D. esculentum* from Central Kalimantan showed the presence of similar compounds, including terpenoids [234]. Biological studies have shown that this plant has anti-bacterial, anti-malarial and larvicidal activities [231,235,236]. The use of *D. esculentum* as food is very popular in Borneo [237], where the fronds are usually eaten as salad or cooked among the Dusun community [238]. The Dayak people in Central Kalimantan use this vegetable as a good source of iron and folic acid, where it is suggested for childbearing mothers [239], while the people of Sarawak usually cook this vegetable with shrimp paste or anchovies [169].

### 5.3. Borneo Wild Ginger

Borneo wild ginger (*Etlingera coccinea*, Figure 4), also known as “tuhau” (Sabah), “tepus” or “tubu nanung” (Sarawak and Brunei), is a plant indigenous to the rainforests of Borneo [141,217,218], particularly in Sabah and Sarawak, Malaysia [240,241,242]. Known for its strong aroma and distinctive flavour, “tuhau” has been traditionally used by indigenous communities in their diets and as a form of natural medicine. The plant’s stems and shoots are most often consumed and are usually prepared as pickles or cooked as a vegetable. As a member of the ginger family (*Zingiberaceae*), tuhau shares several beneficial phytochemicals with ginger, but its unique flavour and nutrients make it a prized ingredient in local cuisine.

Tuhau contains a range of nutrients and bioactive compounds, such as vitamin C; minerals such as K, Ca and Fe [167,243]; and high concentrations of polyphenols, flavonoids and essential oils such as eugenol and pinene, which combat oxidative stress [178,243,244]. It also provides fibre that aids in digestion and helps regulate blood glucose [245,246], and it contains bioactive compounds including gingerols, shogaols, terpenoids, alkaloids and phenolic compounds that exhibit anti-inflammatory and anti-microbial properties [244,247,248,249]. The reported medicinal uses of tuhau include treatment for stomachache, gastric problems, food poisoning, cough and wounds, and it also shows anti-microbial, anti-inflammatory, antioxidant and anti-depressant properties [241,247]. Tuhau’s combination of nutrients and bioactive compounds makes it a valuable part of the traditional Borneo diet and a functional food with potential health benefits for those who incorporate it into their meals.

### 5.4. Torch Ginger/Bunga Kantan

“Bunga kantan” (*Etlingera elatior*, Figure 4) is a tropical flower native to the rainforests of Southeast Asia, including Borneo and the Malay peninsula. This vibrant pink flower, commonly referred to as the “torch ginger” or “ginger flower”, is a staple in local cuisine and is highly valued for its unique flavour and aromatic qualities. Bunga kantan is often used in traditional dishes, imparting a distinct fragrance and taste that enhances various culinary preparations. Its culinary versatility extends beyond being a mere garnish, as it is included in soups, salads and other dishes, showcasing its importance in Bornean and Malaysian cooking. Bunga kantan is appreciated for its culinary uses and also contains several nutrients that contribute to its health benefits, such as vitamins A and C, minerals (Ca, K) and other phytonutrients such as flavonoids and phenolic compounds [250]. In short, the unique flavour and fragrance of bunga kantan enhance the taste of dishes, promoting the consumption of fresh and healthy ingredients.

### 5.5. Green Leafy Vegetables (Ulam)

**Figure 5:**
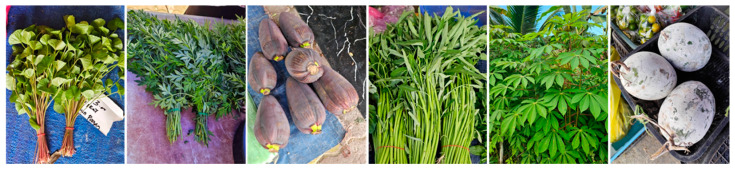
Additional vegetables from Borneo. Left to right: pegaga (*Centella asiatica*), ulam raja (*Cosmos caudatus*), jantung pisang (banana blossom, *Musa* spp.), kangkong (*Ipomoea aquatica*), cassava (*Manihot esculenta*), winter melon (*Benincasa hispida*).

“Ulam” refers to a variety of traditional edible herbs and leafy vegetables commonly consumed raw or lightly blanched in Borneo, often accompanied by sambal (chilli paste) or other local condiments. These wild herbs are an integral part of indigenous diets across Borneo due to their accessibility, unique flavours and rich nutrient profiles. Among the most popular ulam are “pegaga” (*Centella asiatica*, Figure 5), “ulam raja” (*Cosmos caudatus*, (Figure 5), “kacang botor” (*Psophocarpus tetragonolobus*), “petai” (*Parkia speciosa*, Figure 4) and “jantung pisang” (*Musa* spp., Figure 5) [251].

Also known as “gotu kola”, pegaga is a small, leafy green herb with a mild, slightly bitter taste that is renowned for its medicinal and health benefits [252]. Ulam species such as pegaga have been consumed by indigenous Bornean communities for generations, not only for their flavours but also for their therapeutic value. Pegaga is rich in vitamins A, C and B group, which support immune function, skin health and metabolic processes [253]. It also contains important minerals such as Ca, Fe and Mg, which are essential for bone health, blood formation and muscle function [252]. Pegaga and other ulam are excellent sources of dietary fibre, promoting digestive health and aiding in weight management, as well as of antioxidants and bioactive compounds such as phenolic compounds, flavonoids and triterpenoids that have antioxidant, anti-inflammatory and neuroprotective properties [251,253].

“Ulam raja” (or king’s salad), *Cosmos caudatus*, is an aromatic herb that has a slightly tangy flavour and is rich in antioxidants, vitamin C and polyphenols [251]. It is traditionally believed to aid in digestion, support bone health and reduce blood glucose concentrations. “Kacang botor”, *Psophocarpus tetragonolobus* or winged bean, is rich in protein, fibre and vitamins, especially vitamin A and Fe. Its seeds, leaves and pods are all edible, and it promotes heart health and improved digestion [251]. “Petai”, *Parkia speciosa* or stink bean has a distinct, strong flavour and aroma. It is high in dietary fibre, vitamins and minerals, particularly P and Fe. Petai is believed to support kidney health and has anti-bacterial and detoxifying effects [251]. “Jantung pisang” (or banana blossom), banana flower, is normally boiled first before eaten as ulam. They are a good source of fibre, K and antioxidants. Banana blossoms are believed to help regulate blood glucose, promote digestive health and reduce inflammation [251].

Other popular leafy vegetables in Borneo include the “sayur manis”, known as *Sauropus androgynus* (Figure 4), which is usually sauteed with garlic, chilli and eggs [254]. This vegetable is high in protein and vitamin C, making it a good source of protein for the plant-based diet [255]. The plant is also rich in phytochemicals such as solanesol, squalene, phenolics and flavonoids, which impart various health benefits [256]. Despite this, the vegetable is not to be eaten raw, as it contains compounds that may cause bronchiolitis [257]. Wild watercress, *Nasturtium officinale* (Figure 4), another popular green in Sabah that is rich in glucosinolates, is usually cooked stir-fried or lightly blanched and has the potential for further commercialisation [258]. *Crassocephalum crepidioides* (Figure 4), locally known as “tanduk manggarang” by the Bajau people and “gipun” by the Dusun people, is an underutilised yet edible weed commonly found in Kota Belud, Sabah. Sama Bajau villagers traditionally source this plant from local markets or by foraging in forests [259]. The wild Borneo chive, or “losun” (Figure 4), is also popular and iusually mixed with torch ginger and tuhau, making it a unique local ulam combination [260].

The water spinach (*Ipomoea aquatica*), also known as “kangkong” (Figure 5) or water convolvulus, is also commonly found and consumed in Borneo [261,262]. The leaves and tender shoots are usually fried with garlic and chilli paste to make it into a spicy stir-fry dish, often mixed with anchovies or dried shrimps. There are several cultivars of kangkong, of which some can be planted in soil or grown by streams or ponds. Apart from dietary consumption, reports have shown the anti-cancer, hepatoprotective and hypoglycaemic activities of the plant extracts [263,264,265]. Phytochemical analysis showed that the plant extracts contain polyphenols, particularly caffeic acid, ferulic acid, quercetin and rutin [261]. Proximate composition showed the presence of various minerals such as P and K, while the amino acid profile indicated the presence of essential amino acids including lysine, phenylalanine and isoleucine [261].

Ulam and other leafy vegetables are easy to cultivate and grow abundantly in the wild, making them a sustainable food source in rural areas. These plants are valued for their nutritional content, including vitamins, minerals, fibre and potent phytochemicals [252,253]. They are traditionally believed to support longevity, enhance vitality and promote overall wellness, making them a fundamental part of Borneo’s food culture and an emerging focus in nutrition and health research. The biological and medicinal activities of other local vegetables and ulams have been previously reviewed and serve as a valuable additional reference for further information [34,143,237,266,267,268].

### 5.6. Rebung

“Rebung”, or bamboo shoots (*Dendrocalamus* and *Bambusa* spp.), are the edible young shoots of bamboo plants that have become a popular traditional food throughout Borneo, Peninsular Malaysia and other parts of Southeast Asia. Often harvested when they are tender and about 6–12 inches tall, bamboo shoots have a mild, slightly sweet taste and a crisp texture, making them a versatile ingredient in the local cuisine. They are commonly cooked in stir-fries, soups and curries or fermented and preserved for later use. In Borneo, bamboo shoots hold cultural significance as they are available throughout the year and serve as a sustainable food source that is widely harvested from forests, making them an essential part of traditional diets and culinary practices [269].

Bamboo shoots are low in calories but packed with essential nutrients that offer numerous health benefits. Key nutrients found in rebung include B vitamins, such as niacin (B_3_), riboflavin (B_2_) and vitamin B_6_, which play vital roles in energy metabolism, brain function and skin health. They also contain vitamin C, which supports immunity and antioxidant defence. Rebung is rich in minerals such as K, P, Mg and Ca, which are essential for maintaining heart health, muscle function, bone density and cellular processes [270]. Rebung contains a high amount of dietary fibre, which aids in digestion, promotes satiety and helps manage blood glucose concentrations. Rebung contains amino acids that contribute to protein intake, which is particularly beneficial in plant-based diets. Rebung contains phenolic compounds, which have antioxidant properties that protect cells from damage and reduce inflammation in the body [269,270].

### 5.7. Tubers, Stems and Leaves from Yams

Locally known as “keladi”, *Colocasia esculenta* (Figure 4) and *Alocasia macrorrhizos*, are popular traditional vegetables in Borneo cherished for their versatility in cooking and their role in indigenous diets [137,271,272]. In Borneo, indigenous communities such as the Kadazandusun in Sabah often incorporate the tuberous root, young leaves and stems in traditional dishes. These are frequently cooked with pickled or preserved meat or fried alongside other ingredients, reflecting a strong cultural heritage that ties food to social practices [273]. The mild, earthy flavour of keladi is particularly valued and its nutritional benefits contribute to its reputation as a sustainable, nutrient-dense food source, especially in rural regions where resources may be scarce [271,274].

“Ubi gadong” (*Dioscorea* spp.) provides a variety of nutrients essential for health, including carbohydrates, dietary fibre, essential vitamins and phytochemicals [274]. Dietary fibre from these plants aids in digestion, while their complex carbohydrates are a key source of energy for communities with limited access to other food sources. Furthermore, ubi gadong species are rich in micronutrients such as K, Ca and P, which support heart health, bone density and cellular functions [275]. These nutritional attributes make ubi gadong not only a staple food but also a functional food with the potential to support health beyond mere sustenance [272].

The bioactive compounds in keladi, such as phenolic compounds and flavonoids, provide antioxidant benefits that may help reduce inflammation and protect against chronic diseases [271,274,276]. Such properties are particularly beneficial in traditional diets, where indigenous communities may rely on local plants not only for nutrition but also for preventive health measures. In addition, keladi has shown potential for promoting food security due to its resilience and adaptability in varying environmental conditions [273].

The therapeutic potential of these plants has also gained scientific attention. Compounds in *C. esculenta* and *A. macrorrhizos* could contribute to health improvement, supporting their historical use in traditional medicine [271,272,275]. For instance, some communities use keladi extracts for their purported anti-inflammatory and immune-boosting properties, which modern research has started to validate [274]. This combination of nutritional and therapeutic attributes strengthens the role of keladi and ubi in the traditional diets and health practices of indigenous populations in Borneo.

### 5.8. Edible Mushrooms

Borneo is also a thriving spot for wild edible mushrooms due to its humid tropical climate. In Sabah, the wild mushrooms usually consumed by the local populations belong to two major classes of fungi, which are *Ascomycota* and *Basidiomycota* [277]. Among the 25 wild edible mushrooms, only 5 types were reported for their medicinal use: *Pleurotus tuber-regium* (“kulat ubi”), *Xylaria* spp., *Auricularia* spp. (“kulat telinga”), *Schizophyllum commune* (“kulat kodop”) and *Lignosus* spp. (“cendawan susu harimau”) [277]. *Xylaria* spp. are also reported in Kalimantan for their culinary and medicinal uses [278]. *S. commune* is the most well known and is usually seen at local markets in Sabah (Figure 4) [277]. These mushrooms are usually eaten on their own or incorporated into traditional dishes with meat or local vegetables or other mushrooms. The local Dusun community has been using *S. commune* with *E. coccinea* to prepare special floss called “serunding” [277]. In an earlier analysis, *Auricularia* spp. have the highest amounts of Mg, *Pleurotus* spp. of Ca and *S. commune* is rich in K [279]. As for medicinal usage, *Lignosus* spp. have been used for cough treatment and wound healing; *Xylaria* spp. for making a wrist band for health; and *S. commune*, *Auricularia* spp. and *Pleurotus tuber-regium* were used for making soups to treat fever and cold [277]. Despite this, caution is still needed when selecting wild mushrooms for consumption and poisoning cases still occur from time to time [280].

### 5.9. Staple Starch-Based Food

The main dish of Borneo includes a starchy food as a source of carbohydrate. Special varieties of rice exist in Borneo, which have been increasingly studied for their phytochemical and health properties. Mayas and Adan rice are among the native highland rice varieties in Borneo cultivated for hundreds of years [281]. In Kalimantan, Adan rice are some of the premium varieties, which consist of the white, red and black varieties. The highest Fe content was found in white Adan rice, with all five varieties studied having vitamin B_1_. Black Adan rice has the highest insoluble dietary fibre and protein content and the lowest glycaemic index, shown by the slowest blood glucose spike in study subjects [281]. Similarly in Sabah and Sarawak, the upland rice varieties are widely cultivated for personal consumption, but production is increasing to meet growing demand [282]. The various types of rice in Sabah and Sarawak have been officially reported recently [283]. The upland rice varieties “Bario” and “Bukit Pulut” showed notable antioxidant and phytochemical properties. The Bario rice contains the highest total phenolic and total flavonoid contents with higher ferulic acid, salicylic acid and *p*-coumaric acid compared with the other tested rice varieties [284]. Of particular interest is the presence of pigmented rice, also called “tadong” rice (*Oryza sativa* var. Tadong, Figure 4) in Sabah, showing higher anthocyanin contents compared with other varieties of Sabah’s upland rice [285]. Pigmented rice grains have superior nutritional and phytochemical properties, including flavonoids and anthocyanins, with anti-diabetic, anti-cancer, anti-obesity and renal protective properties, and they could direct towards healthier gut microbiota composition [27,286,287,288,289]. A traditional wrapped-rice dish called “linopot” is popular in Sabah and has gained popularity both among locals and tourists. The dish is prepared by wrapping upland pigmented rice with local leaves such as tarap, banana or “wonihang” leaves, which also give unique flavours to the rice [290]. The rice is usually packed with fish or meat, pickled and fermented foods, and other vegetables, and it is often cooked together with yams, sweet potatoes (*Ipomoea batatas*, Figure 4) or pumpkins, which infuse to give it a purplish colour [290,291]. Looking into the current literature, there is a lack of reports detailing the biological activities of pigmented foods from Borneo, and this calls for more experimental studies to further explain their beneficial health properties.

Another popular staple food is sago from the tree *Metroxylon sagu*. Sarawak is presently the largest sago starch producer in Malaysia [292]. The white starchy powder of the ground inner bark of the sago tree is turned into a sticky paste by slowly heating it with water; it becomes slightly translucent when cooked. The cooked sago starch paste, known as “ambuyat”, is a national dish of Brunei and is usually eaten with several side dishes such as curries, fish, and spicy and sour gravy [293]. The dish is usually eaten as an alternative to rice, and it was often credited to giving sustenance during challenging times [294]. Ambuyat is also popular in Sabah, where it is eaten among the Bisaya, Dusun-tatana and Brunei-Malay tribes [295]. For the Melanau tribe in Sarawak, sago pellets have been used as a staple food eaten as snacks or side dishes [296]. Another species, *Eugeissona utilis*, the Borneo sago palm, is used by the Sarawakian Penan community to make sago starch, which has a deep cultural significance [297]. Other potential applications of sago include its use as an ingredient for making noodles, biscuits, cakes and even sago sugar due to its high carbohydrate content [298]. The sago plant, while still understudied, serves as an important alternative to address food security issues in the future [299].

### 5.10. Root Vegetables

Palm sugar, “gula semut” or “gula apong” is a natural sugar made from palm sap by dehydrating the palm sap, which is rich in glucose and sucrose, through cooking, followed by cooling and crystallisation, resulting in a brownish sugar product [300]. There are several varieties of palms that produce sweet palm sap for sugar production, such as the *Arenga pinnata*, *Nypa fruticans*, *Borassus flabellifer* and *Cocos nucifera* palms [301]. Palm sugar from *Argena pinnata* has a low glycaemic index, shown by the slow blood glucose increase after consumption [302], along with probiotic properties [300]. The sugar from the *Nypa fruticans* palm is widely known as “gula anai” in Brunei or “gula apong” in Sarawak, where it is reported to have low glycaemic index properties and good amounts of phytochemicals (flavonoid and phenolic compounds), vitamins and minerals [303,304]. The presence of phenolic compounds such as chlorogenic acid, cinnamic acid and kaempferol suggests that the sugar has good antioxidant properties [305]. Apart from sugar, the fresh palm sap can also be fermented to produce palm vinegar [306].

Cassava plants (*Manihot esculenta*, Figure 5) are also widely consumed in Borneo, where the leaves, also known as “daun ubi” or “daun singkong”, are cooked as vegetables or used in fermented products [167,239]. The starchy roots or tapioca are eaten as a replacement for rice and other carbohydrates [307]. Nutritional analysis showed the content of essential minerals such as Ca, P, Mg and K, and vitamins A, B, K and C [308]. The medicinal uses of the pulp paste include using it as poultice to alleviate headache, the combination of honey and the leaves’ juice for treating constipation, and the fresh juice used for healing haematemesis [308]. The leaves and roots are increasingly used to make a variety of food products including cakes, biscuits and cassava chips, and the leaves are used for stir fry and soups [309,310,311]. The leaves are reported to have several biological activities including anti-cancer, anti-inflammatory, anti-diarrheal, anti-bacterial and wound healing activities [308]. However, caution should be taken against excessive consumption of tapioca and the leaves, as they are reported to contain anti-nutrient cyanide compounds [307,312].

### 5.11. Other Vegetables

*Archidendron pauciflorum*, synonym *Archidendron jiringa*, is also known as “jering” in Malaysia, “jengkol” in Indonesia or “luk nieng” in Thailand [313]. The jering beans are usually eaten raw, fried or roasted and are commonly consumed in Southeast Asia, including Borneo [313,314,315]. The plant was reported to have anti-microbial, antioxidant, anti-cancer, anti-gastric and anti-nematodal properties [313]. Care should be taken when consuming jering beans as toxicity cases, termed djengkolism, have been reported. Djengkolism is often linked with the presence of nitrogenous compounds and the hypersaturation of djenkolic acid crystals in the urinary system, leading to acute anuric renal failure [313,316].

Among the gourd family, the winter melon (Figure 5) or winter gourd (*Benincasa hispida*) also known as “kundur”, “labu putih” (Malay) or “tonsomon” (Kadazandusun) is also widely used for making soup dishes in Borneo [317,318]. As a vegetable, the fruit provides amino acids, organic acids, minerals, vitamins and natural sugars [319] along with various phytochemicals such as flavonoids, sterols, glycosides, phenolic compounds, steroids and fats [318]. The ethnomedicinal uses of winter melon include usage to treat cough with thick mucus, fever and urinary disease [320]. It is also known for its cooling, laxative and diuretic properties and for alleviating menstrual disorders, among others [319]. The pharmacological effects reported include antioxidant, anti-inflammation, cytotoxic, lipid-lowering, neuroprotective, bronchodilator, anti-hypertensive, nephroprotective and skin protective effects [320]. The winter melons are grown locally and often sold in the weekly Sunday markets.

### 5.12. Other Plant-Derived Food Sources

The demand for native honey as a healthier alternative for commercial sugar is gradually increasing [321]. Honey is an energy-rich product produced by a variety of bees, including honeybees and stingless bees [322]. Stingless bee honey is widely used in Borneo; for instance, a report showed that *Geniotrigona thoracica* honey has high flavonoid contents and anti-bacterial properties, while honey of *Heterotrigona itama* has high antioxidant properties and flavonoid contents [322].

## 6. Incorporating Bornean Fruits and Vegetables into Other Asian and Western Diets

The consumption of fruits from Borneo can be enhanced through various processing to enhance acceptability and attractiveness; for instance, *M. pajang* fruits can be turned into fruit juice for longer shelf life, transportability and marketability [323]. A recent study reported that bambangan kernel fat is suitable for use as a cocoa butter replacement in foods, especially confectionary products [324]. The other Bornean tropical fruits such as mangosteens, durians, rambutans and salaks could also be utilised for making other products such as ice creams, sorbets, salad dressings and as constituents for desserts, biscuits and cakes. Fruit salads including rambutan as ingredients are one of many options. Mangosteen is a versatile fruit that can be enjoyed in a variety of ways. It can be eaten fresh, added to fruit salads or juiced. It is a popular ingredient in desserts such as ice cream, sorbet and clafouti. Additionally, mangosteen can be used as a garnish for puddings such as Thai tapioca pudding. For a unique twist, mangosteen also can be added to savoury dishes to add a sweet and tangy flavour profile. Durians, other than fresh consumption, can be used in cakes, candies, pastries and ice creams. Their application for making mooncake fillings is also widely popular. Mixing durian with milk or coconut milk, sugar and ice makes a delightful creamy treat. The durian’s creamy and sweet flesh is a unique ingredient for making fillings for crepes and cakes [325]. As a healthier alternative, palm sugar can be used to replace sugar for the production of desserts and drinks. Other fruits can also be made into pies, creams, custards and fritters. The suggestions for the vegetables’ application into the Western diet are also plenty; for instance, the various leafy green vegetables can be added to salads or pesto or used as a garnish for soups and stews. Midin, for example, can be stir-fried with garlic and butter or added to pasta dishes, or sayur manis can be roasted or grilled and served with a dipping sauce and used as filling for sandwiches. Terung dayak can be used together with eggplants for making ratatouille or roasted and served with a balsamic glaze. These are several examples of culinary uses of Bornean foods and should be modified accordingly to preferences and creativity.

## 7. Opportunities and Innovations

According to the Food and Agriculture Organization of the United Nations, around 14% of global food production was lost or wasted after harvest in 2019, leading to significant economic losses [326]. In Europe, 50% of the food waste generated by households was contributed by fresh fruit and vegetables, and a large proportion of the waste came from inedible fruit and vegetable parts [327]. The waste parts of fruits and vegetables usually include leaves, pomace, peels, skins, seeds and stubs [328]. Tropical fruits are an important source of dietary fibre, vitamins and minerals. However, the processing of these fruits often generates substantial amounts of waste, including peels, seeds and pulp. Traditionally, these by-products have been discarded as low-value materials. However, recent research has revealed the potential of tropical fruit waste as a valuable source of bioactive compounds with significant health benefits [329]. Addressing tropical fruit waste is not only critical for mitigating environmental impacts but also for unlocking potential opportunities for resource utilisation and value-added products [330]. There are many suggested ways for how the fruits’ and vegetables’ waste parts can be used for valorisation, including as a source of bio-adsorbents, dietary fibre, bioactive compounds, enzymes and energy (biofuels); reusing the waste in various industries such as food, health, textile and chemical; and in wastewater management and renewable energy [328,330].

Bioactive compounds such as phenolic compounds and antioxidants are abundant in various parts of tropical fruits, including the peels and seeds. These compounds have been linked to numerous health benefits, including antioxidant, anti-inflammatory and anti-cancer properties. Studies have shown that the peels of fruits such as mangosteen and mango contain higher concentrations of total phenolics than the flesh itself [329]. Similarly, the peels of papaya and dragon fruit are rich in bioactive compounds [329]. By employing innovative extraction techniques, these bioactive compounds can be efficiently recovered and used in various industries.

In the context of Bornean foods, the peel and seeds of *M. pajang* have been reported to have high amounts of phytochemicals and are considered cheap sources of foods and nutraceuticals [331]. The promotion and use of bambangan fruit waste by-product can be included into food products or used as a sustainable source of dietary fibre. The addition of mangosteen flesh and seeds in cracker production increased several nutrients such as omega-6 and omega-9 fatty acids, fibre, minerals and amino acids [332]. This encourages the usage of the seed as a food constituent. The seed flour is also rich in minerals including K, Mg and Ca [333]. The analysis of the mangosteen seed showed high oil content (22%) with characteristics comparable with other conventional edible oils and suggests its potential use as an edible oil for industrial applications [333]. Furthermore, pigmented vegetables such as the purple long bean (Figure 4), bayam merah and red spinach (Figure 4) could be further explored for extraction of phytochemicals [334].

The analysis of methanolic extracts of two native durian fruit parts showed that the highest total phenolic content was found in the mesocarp of *D. kinabaluensis* (205.16 ± 11.6 mg/gallic acid equivalent/g sample) followed by the exocarp of *D. oxleyanus* (161.03 ± 5.9 g/gallic acid equivalent/g sample), whereas the highest total flavonoid content was found in the *D. oxleyanus* exocarp and the seeds of *D. kinabaluensis*. These parts also showed high antioxidant activities, showing that the non-consumable parts of durians have large potential to be used as sources of nutraceuticals and phytochemicals [39]. The rambutan’s seed has also been studied as a rich source of raw materials providing starch, protein and fat [335], and it has the potential to be used as a cocoa butter improver [73,336].

The unique phytochemical and physicochemical constituents of the indigenous Bornean foods may provide an opportunity for further innovation of plant-based products. For instance, the anti-microbial and antioxidant properties in ferns may be applied for food, cosmetics and food packaging materials [225]. The use of brown seaweed extract as a biostimulant has increased the growth and yield of pigmented upland rice in Borneo [337]. The production of bioplastics from the entire biomass of red seaweed (*Kappaphycus* spp.) with incorporation of natural dyes from butterfly pea flower, bougainvillea flower and dragon fruit peel increased the antioxidant properties and shelf life of the bioplastics suitable for low-tensile food packaging applications [338]. The valorisation of tropical fruit waste offers a sustainable solution to a significant environmental challenge. Transforming waste into valuable resources can promote a circular economy and contribute to a more sustainable future.

Borneo’s tropical climate, characterised by abundant rainfall, high humidity and consistently warm temperatures, provides a conducive environment for a rich diversity of flora and fauna. These favourable conditions support the cultivation of numerous tropical medicinal plants, with around 1300 species identified for medicinal use [339]. Traditional medicine, passed down orally through generations, remains an integral part of healthcare for many communities on this island.

Borneo’s importance extends beyond its geographical attributes, serving as a vital hub for cultural exchange. Known for its rich cultural diversity and unspoiled natural environment, the island is home to numerous ethnic groups and indigenous communities, each preserving unique languages, traditions and healing practices [340]. Traditional healing practices in Borneo encompass diverse modalities such as herbal medicine, spiritual healing and ritual ceremonies [341]. The dense rainforests of Borneo are a treasure trove of medicinal plants with a wide array of therapeutic benefits. For centuries, indigenous communities have relied on these plants to address ailments such as fevers, infections, chronic conditions and even spiritual disturbances [342]. Traditional knowledge in Borneo is deeply interconnected, with indigenous communities often sharing practices and utilising common plant species [343]. This interdependence underscores the need for collaborative efforts to document and safeguard traditional knowledge for future generations.

Sabah is renowned for its ethnic and biological diversity, but ethnobotanical knowledge from East Malaysia remains relatively underexplored [344]. Interest in documenting ethnobotanical practices in Sabah surged during the late 1980s and early 1990s, largely driven by the need to record plant resources amid the rapid expansion of the logging industry. One notable initiative, the Kinabalu Ethnobotany Project of the 1990s, recorded 168 plant species used by the Dusun community of Kinabalu. Research in Sabah has predominantly focused on indigenous groups such as the Kadazandusun, Murut, Idaahan, Orang Sungai, Bajau and Illanun, highlighting their deep connection with the forest ecosystem [137,268,344,345,346,347,348].

## 8. Limitations in the Current Studies

The production of plant-based foods in Borneo faces a range of interconnected challenges that could affect the availability and sustainability of these resources. There are several issues and limitations that need to be addressed to ensure continued supply for consumers. The supply of some tropical fruits may not be regular due to fruiting seasons [349,350,351]. Thus, this will limit the production and cultivation to plants that yield products throughout the year. Excess rainfall and drought can affect production and growth, leading to decreased harvests and supply, as may climate change. The inconsistencies in production may affect food security in the long run but also create a variable demand in the market for seasonal fruits, hence resulting in fluctuating market prices.

It is difficult to preserve tropical fruits and vegetables due to limitations in storage and preservation facilities, especially in the interior part of Borneo where infrastructure may still be incomplete. As many fruits and vegetables have short shelf lives, the local produce from remote villages may not be transported in a timely manner to the consumers, leading to post-harvest losses, waste and financial losses [352]. Traditional preservation measures such as fermenting, salting, drying and smoking may be used, but these are labour-intensive methods and change the texture, taste and nutritional content of the food products. The deforestation of the land mass in Borneo due to plantation establishment and expansion, logging and urban land conversion is another aspect to be considered [7,353]. A balanced decision should be observed between using land for economic purposes and preserving and maintaining biodiversity and local ecosystems. The decreased forest cover can impact the cultivation of non-commercial crops that are integral to local diets [354]. For instance, deforestation and land conversion into crop planting may impact the local fungi population, which has raised concerns among many local conservation biologists [277].

Many local farmers still rely on slash-and-burn techniques for clearing land, which could contribute to soil degradation, loss of biodiversity and forest fires [355,356]. Furthermore, the lack of access to modern agricultural technologies such as irrigation systems, fertilisers and pest control methods limits production. However, this limited intervention might be a positive for consumers seeking organic food. Looking forward, there is a need to explore innovative agricultural practices that increase yield while minimising pollution. This could involve sustainable practices and responsible land management techniques. Another key area for advancement lies in research. In-depth studies are needed to fully understand the biological, chemical and medicinal properties of Borneo’s plant-based foods. Ideally, these laboratory findings should translate into clinically relevant outcomes for the general population. Building such a foundation of scientific evidence can reduce public doubt and bias towards these unfamiliar traditional foods, ultimately encouraging their consumption.

The search for alternative foods whose sustainable production and high nutritional quality guarantee regular access to food for the population must be encouraged. Alternative foods can contribute to food security in many ways as they contribute to the local economy and income generation [357]. One of the challenges is to demystify and popularise the uses of unconventional plant-based foods, ancestral grains and flowers [358], and to implement sound food policy practices effectively [359]. Novel product development opportunities with these native Bornean foods for commercial purposes will require the evaluation of many aspects, including various consumer acceptance aspects [360]. Further areas to consider before and during the commercialisation include potential sustainability challenges such as sustainable cultivation methods, overharvesting, habitat destruction and climate change impacts on biodiversity.

## 9. Conclusions

This review highlights the rich diversity and nutritional potential of plant-based foods from Borneo. These foods are abundant in bioactive compounds with promising health benefits, including antioxidant, anti-inflammatory and anti-microbial properties. Their unique compositions underscore their potential for both traditional and modern applications in the food and health industries. Despite the promising potential of Bornean natural foods, several key gaps in the research must be addressed to unlock their full benefits.

While traditional knowledge and preclinical studies highlight the health benefits of Bornean plant-based foods, there is a lack of robust clinical trials on human subjects. For example, fruits such as bambangan and purple mangosteen, widely known for their medicinal properties, are usually studied using *in vitro* or *in vivo* models. However, comprehensive human trials are required to validate their efficacy, establish safe dosages and understand potential side effects. Such trials could also explore the long-term effects of incorporating Bornean fruits and vegetables such as durian, dabai, seaweeds, ulams and fermented foods into human diets, focusing on chronic diseases affecting cardiovascular and metabolic health.

The FAO White/Wiphala paper on indigenous foods discusses the role of the food systems of indigenous peoples, especially in the debate on sustainable food resources, as well as the drivers affecting these food systems [361]. The challenges in food security in the ASEAN countries, including Malaysia, Brunei and Indonesia need an integrated approach to overcome hurdles such as climate change and land degradation through soil erosion, deforestation and unsustainable farming practices [362]. However, the transformation of Bornean natural foods in this review into value-added products such as functional foods and nutraceuticals remains underexplored. Value-added plant-based foods and therapeutics could include *Etlingera elatior* (torch ginger), wild ginger such as tuhau, wild berries, ulams and root vegetables known for their antioxidant and anti-inflammatory properties after processing into herbal teas, dietary supplements or fortified snacks. Similarly, oils extracted from indigenous nuts such as dabai and engkabang and seeds such as jackfruit and tarap seeds could be marketed as premium cooking oils or skincare products. Extracts from fruits such as langsat, liposu and winter gourds have also been reported for their skin-protecting effects and may be further commercialised for skin-care products. Fruit waste parts such as purple mangosteen peel, bambangan peel and seeds, durian peel and seeds and jackfruit peel produce large amounts of waste; nutraceutical extractions from these resources using efficient, cost-effective and environmentally friendly processes could generate considerable income for entrepreneurs [363,364]. Such innovations would not only boost the market value of these resources but also provide consumers with convenient and effective ways to improve health by incorporating them in their diet.

Processing methods such as drying, boiling or fermentation can impair the stability and efficacy of the bioactive compounds in Bornean foods. For example, heat-sensitive antioxidants in durians, mangosteens, dabai and local vegetables may degrade during traditional drying processes, reducing their nutritional value. Research should investigate modern preservation techniques, such as freeze drying or vacuum sealing, to retain these components. Additionally, fermented products such as those made from pangium, bambangan or tempoyak could be studied to understand how fermentation enhances or modifies their probiotic and health-promoting properties.

Addressing these research gaps with practical examples and innovative approaches could realistically achieve the potential of Bornean plant-based foods to contribute to global nutrition, health and sustainability. This will not only examine traditional uses but also create new opportunities for scientific discovery, improved health and economic growth.

Overall, Borneo’s rich variety of plant-based foods demonstrates the island’s high potential as a hub for diverse, sustainable and nutritious food sources. These foods are not only integral to the local cultures and communities but also offer many health benefits that could contribute to food security both locally and globally. With focused efforts on strategic commercialisation, cultivation, processing and preservation of foods, Borneo could become an important player in both the regional and international food systems. Further, as the world is increasingly exposed to the consumption of plant-based diets for health, ethical and environmental reasons, Borneo could tap into this opportunity to supply unique plant-based foods to the wider global market. In conclusion, through concerted actions for preservation, production, investment, innovation, continuous research and careful management, the island’s plant-based foods have the potential to contribute to both local and global food sustainability and security.
